# Tocilizumab in the treatment of hyperferritinemic syndrome and capillary leak syndrome secondary to rheumatoid arthritis: Case report and literature review

**DOI:** 10.1097/MD.0000000000038104

**Published:** 2024-05-10

**Authors:** Zhendong He, Hanyou Mo, Leting Zheng, Wen Zeng, Jing Wen, Zhanrui Chen, Fang Qin

**Affiliations:** a Department of Rheumatology and Immunology, First Affiliated Hospital of Guangxi Medical University, Guangxi Zhuang Autonomous Region, P. R. China

**Keywords:** capillary leak syndrome, rheumatoid arthritis, the hyperferritinemic syndrome, tocilizumab

## Abstract

**Introduction:**

Rheumatoid arthritis (RA) is a chronic systemic autoimmune disease, which is mainly characterized by joint swelling, pressure pain and joint destruction. Some patients may suffer from a variety of serious complications, which require prompt diagnosis and treatment. Otherwise, the patient condition may deteriorate rapidly, leading to premature death.

**Objective:**

We reported a case of RA combined with hyperferritinemic syndrome and capillary leak syndrome (CLS) that was successfully treated with tocilizumab (TCZ), with the aim of improving diagnostic ideas for clinicians and consequently improving the diagnosis and treatment of the hyperferritinemic syndrome and CLS.

**Case summary:**

A 55-year-old female patient was admitted to the Department of Infectious Diseases of our hospital due to “recurrent fever for more than 1 month and aggravation for 3 days.” The patient was diagnosed with fever of unknown origin (lung infection?) and received anti-infective therapy with large encirclement of anti-bacterial, antifungal and empirical anti-tuberculosis successively during hospitalization in the Department of Infectious Diseases. Yet her condition continues to progress. The patient was eventually diagnosed with RA combined with hyperferritinemic syndrome and CLS. Then she received glucocorticoids (GC) (160 mg qd) combined with intravenous immunoglobulin (IVIG, 20 g/d, for 3 days). We considered that the patient also had an overwhelming proinflammatory cytokine storm, so she received a strong anti-inflammatory treatment with TCZ (400 mg qm). The patient symptoms and follow-up chest CT showed significant improvement following treatment.

**Conclusion:**

TCZ has good efficacy in the treatment of RA combined with hyperferritinemic syndrome and CLS and is expected to be a promising treatment.

## 1. Introduction

In the real-world clinical setting, we rarely encounter cases of rheumatoid arthritis (RA) combined with hyperferritinemic syndrome and capillary leak syndrome (CLS). These patients always had an urgent onset, severe systemic symptoms, and critical condition. Here, we reported a case of RA combined with hyperferritinemic syndrome and capillary leak syndrome that was successfully treated with tocilizumab.

## 2. Case

A 55-year-old female patient was admitted to the Department of Infectious Diseases of our hospital on October 2, 2022, due to “recurrent fever for more than 1 month and aggravation for 3 days.”

On August 20, 2022, the patient developed an unprovoked fever. Predominantly low-grade fever, occasionally exceeding 39.0°C, accompanied by coughing and coughing a small amount of sticky white sputum. Chest computed tomography (CT) examination in local hospital which showed “lung infection.” The patient was discharged with symptoms relieved after receiving empirical anti-infective therapy. Half a month later, she developed fever again, with temperature fluctuating around 38.5°C. Then, she was hospitalized at a local hospital to complete peripheral blood NGS examination which revealed a low sequence number of *Acinetobacter baumannii* and *Pseudomonas aeruginosa* infections. Doctors reinforced anti-infective regimen but she continued to have high fever with chills and rigors. So the patient came to our hospital.

**Figure 1.**
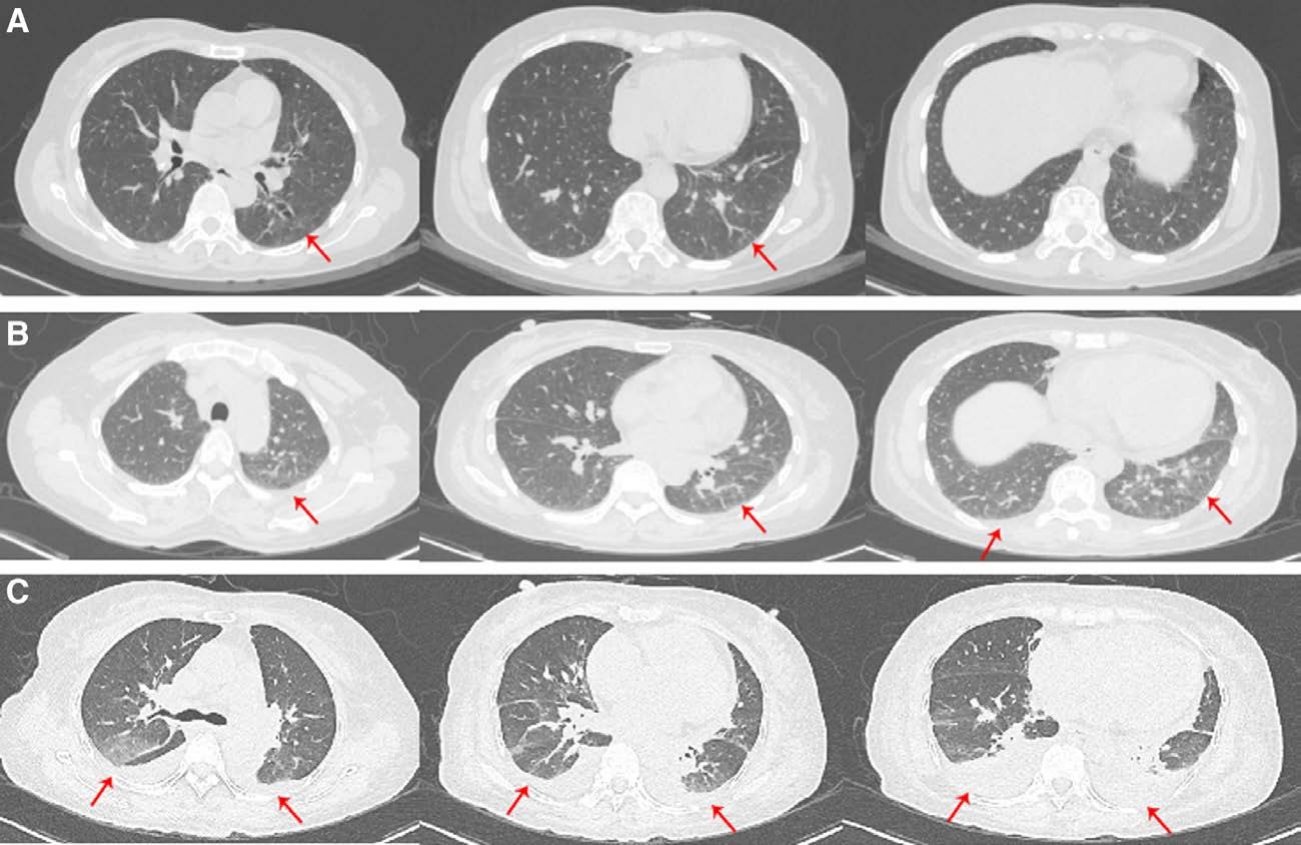
Chest CT images of the patient in the early stages of the disease. (A) CT image of the patient on October 2, arrows showed a few exudative lesions in both lungs. (B) CT image of the patient on October 21, arrows showed slightly increased exudative lesions in both lungs. (C) CT image of patient November 3, arrows showed significantly increased ground-glass lesions in both lungs and new pleural effusion.

**Figure 2.**
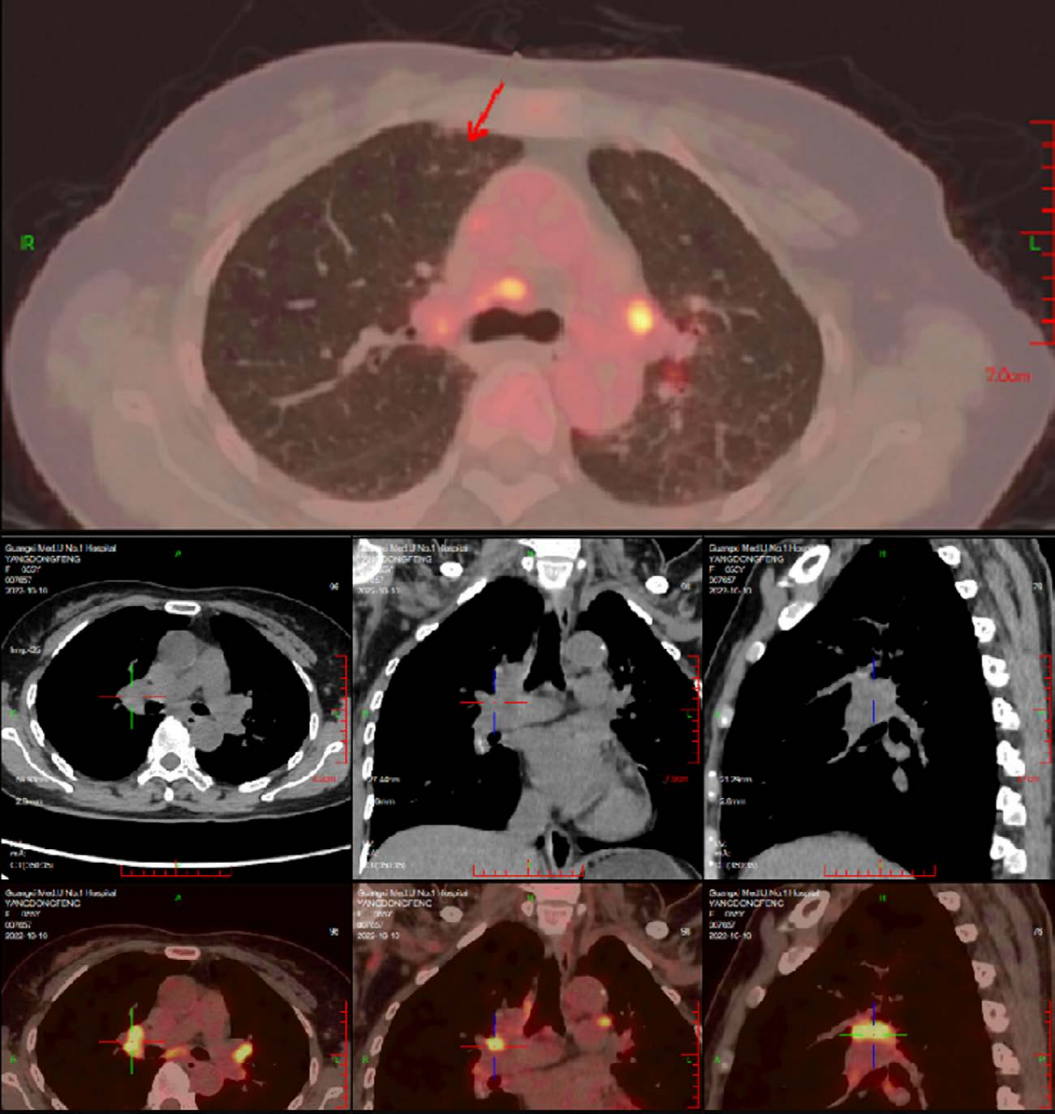
PET/CT images of the patient. Arrows showed inflammatory hyperplasia of multiple systemic lymph nodes (bilateral neck areas I, II, and V, bilateral axillae, mediastinum, bilateral hilum, and bilateral groin) and a slight inflammation in both lungs. PET/CT = positron emission tomography-computed tomography.

**Figure 3.**
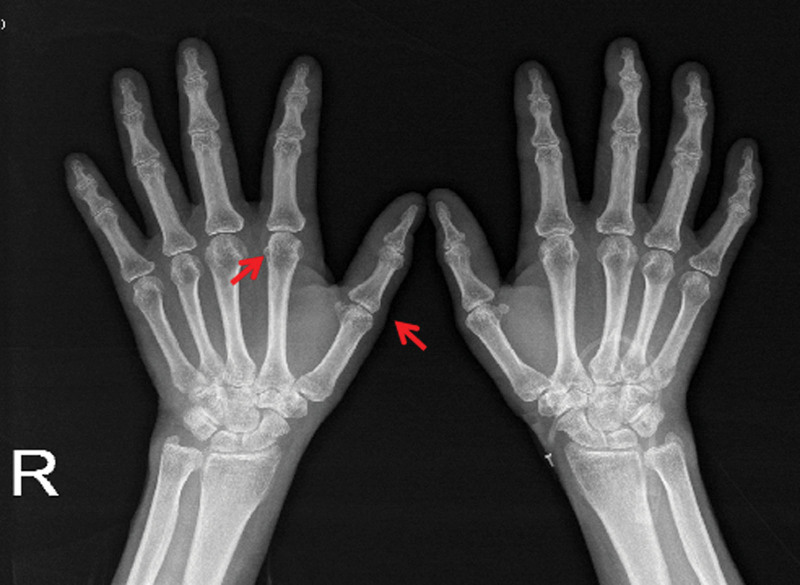
X-ray images of patient hands. Arrows showed osteoporosis of both hands; soft tissue swelling around the proximal interphalangeal joints of both hands and the first metacarpophalangeal joint of the right hand.

**Figure 4.**
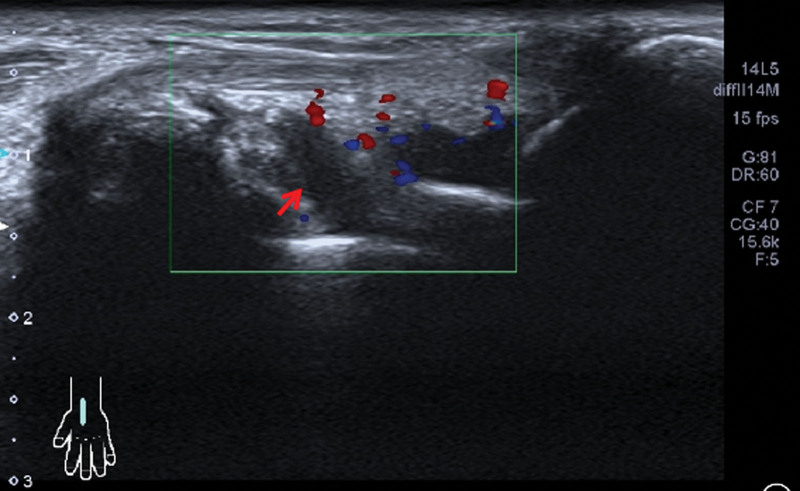
Ultrasound images of the patient wrist. Arrows showed synovial thickening of the synovium of both wrists and a small amount of blood flow signal.

**Figure 5.**
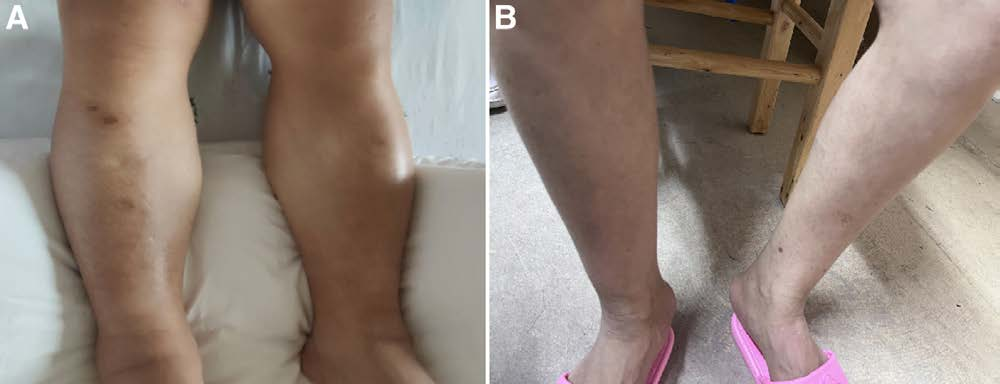
(A) Image of severe edema in both lower limbs before treatment. (B) The edema of the lower limbs resolved after these treatments.

**Figure 6.**
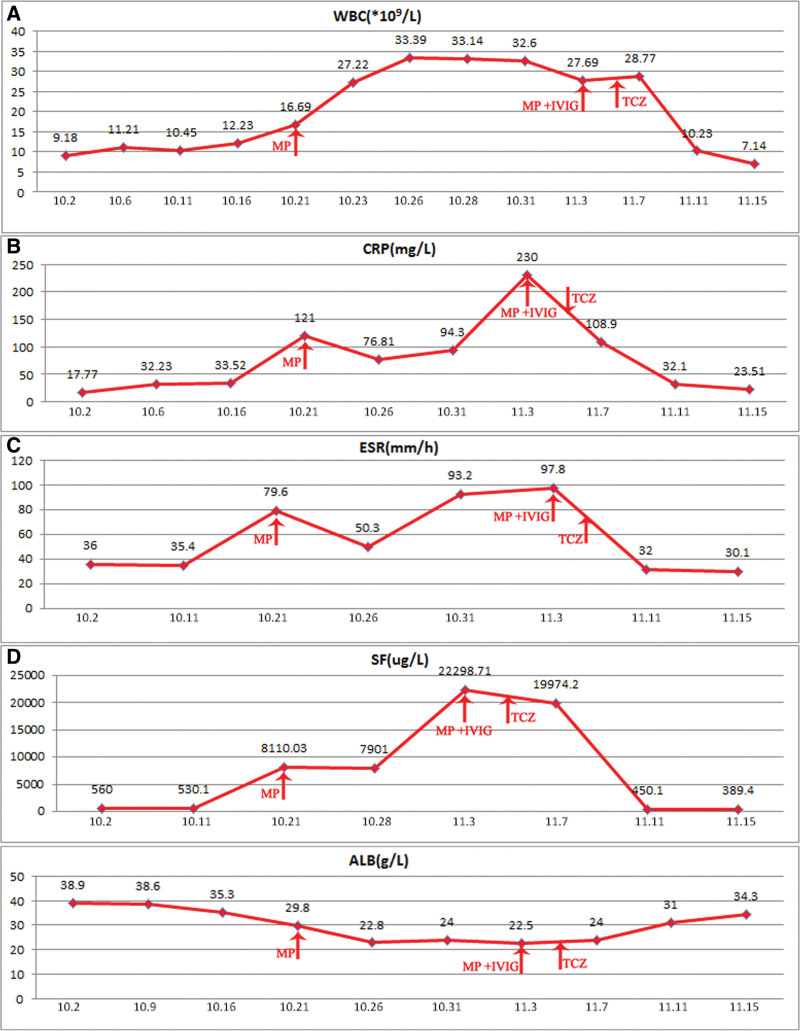
Dynamic changes of peripheral blood reexamination indicators in patients. A showed the changes of white blood cell count before and after treatment. B showed the changes of high-sensitivity C-reactive protein levels before and after treatment. C showed the changes of erythrocyte sedimentation rate levels before and after treatment. D showed the changes in ferritin levels before and after treatment. E showed the changes in albumin levels before and after treatment.

**Figure 7.**
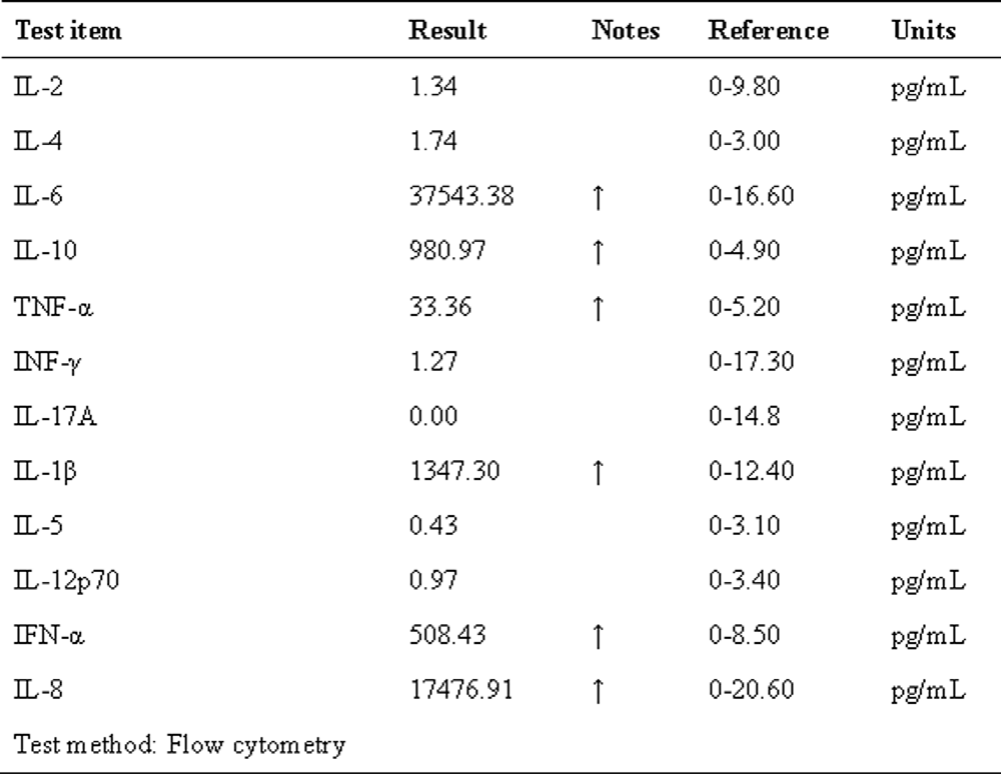
Detection results of peripheral blood inflammatory factors in patients.

**Figure 8.**
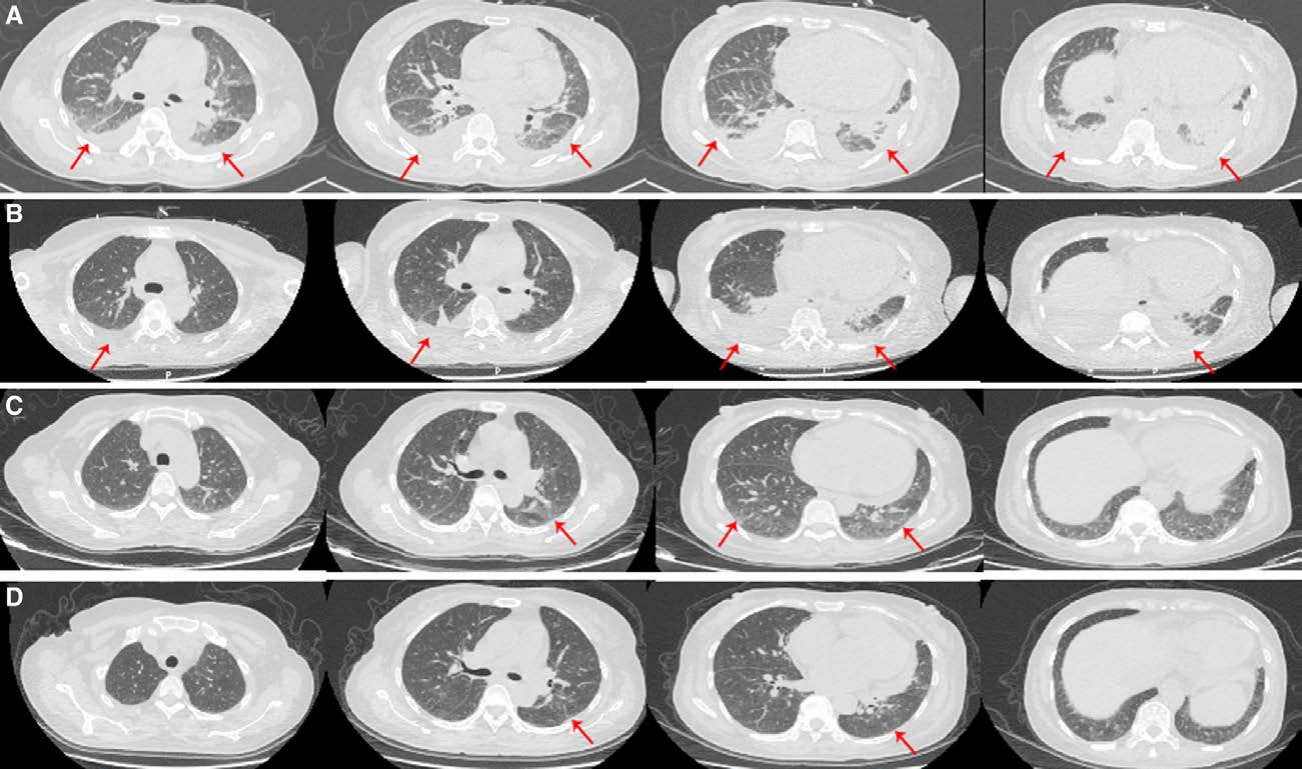
Chest CT images of the patient at later stages of disease. (A) CT image of the patient on November 5, arrows showed a large amount of ground-glass lesions and moderate amount of pleural effusion in both lungs. (B) CT image of patient on November 8, arrows showed a large amount of ground-glass lesions and moderate amount of pleural effusion slightly reduced than before. (C) CT image of patient on November 14, arrows showed significantly less ground-glass lesions in both lungs, and pleural effusion had been absorbed. (D) CT image showed only a small amount of inflammatory lesions in both lungs when the patient came back to hospital for reexamination 1 mo after discharge.

On physical examination, the patient temperature was 38.2°C, heart rate was 92/bpm, respiratory rate was 20 breaths/min, blood pressure was 115/79 mm Hg. The patient had clear consciousness, and a soybean-sized lymph node was palpable on the right side of the neck, which was moderate in quality, mobile, and non-tender. Breathing was slightly coarse in both lungs, and moist and dry rales, as well as pleural friction rub sounds, were not audible in both lungs. Cardiac and abdominal examination of the patient was unremarkable. There was no swelling or pain in the joints of the body, and no edema in the limbs. After admission, the blood work showed the following: white blood cells, 9.18*10^9^/L; hemoglobin, 92.8 g/L; mean corpuscular volume, 59.38; mean corpuscular hemoglobin, 19.54. Her urine routine, stool routine, and blood biochemistry tests were unremarkable. The pathogenic smears and cultures of sputum, peripheral blood, bone marrow, and CSF were all negative. Tumor markers in patients’ serum were all negative. The electrocardiogram was within the normal range and the cardiac ultrasound did not show any abnormalities. Superficial B ultrasound revealed a 1.2 * 1.0 cm lymph node detected in the right neck, and pathological biopsy of the lymph nodes revealed no neoplastic lesions. Chest CT (Fig. 1) examination on admission revealed a few exudative lesions in both lungs. Positron emission tomography-CT (Fig. 2) examination revealed: Inflammatory hyperplasia of multiple systemic lymph nodes (bilateral neck areas I, II, and V, bilateral axillae, mediastinum, bilateral hilum, and bilateral groin). Slight inflammation in both lungs. Bone marrow with increased glucose metabolism, which was likely reactive changes. A bone marrow biopsy showed that proliferation of the bone marrow was active, and abnormal cells were not detected. The patient was diagnosed with fever of unknown origin (lung infection?) and received anti-infective therapy with large encirclement of anti-bacterial, antifungal and empirical anti-tuberculosis successively during hospitalization in the Department of Infectious Diseases. Yet her condition continues to progress. During the treatment, the patient complained of swelling, pain, and stiffness in many joints and particularly the metacarpophalangeal and proximal interphalangeal hand joints, wrists, ankles, elbows, and knee joints. Autoantibody test showed the following: ANA, + (1:320); RF, +; Anti-CCP, +; Anti-ENA, −; Anti-dsDNA, −; Antiphospholipid antibodies, −; C3/C4, −. X-ray examination (Fig. 3) revealed osteoporosis of both hands and minimal soft tissue swelling around the proximal interphalangeal joints of both hands and the first metacarpophalangeal joint of the right hand. Joint ultrasonography examination (Fig. 4) revealed thickening of the synovium of both wrists and a small amount of blood flow signal. The patient was diagnosed with definite RA and transferred to our department on October 21, 2022 for continued treatment. She was treated with glucocorticoids (GC, 40 mg qd) and methotrexate (MTX, 10 mg qw) with a slight decrease in peak body temperature, but still had recurrent fever. On November 03, 2022, the patient presented with worsening condition, characterized by severe cough with chest tightness, progressive worsening of anasarca (Fig. 5), increased pleural effusion and decreased blood pressure, with a minimum blood pressure of 84/44 mm Hg. Dynamic reexamination showed a significant increase in white blood cell count, high-sensitivity C-reactive protein and ferritin levels, and a progressive decrease in albumin levels (Fig. 6). Inflammatory factor test results suggested significantly increased levels of IL-1β, IL-6, IL-8, IL-10, TNF-α, and INF-α (Fig. 7). A repeat chest CT revealed progressive ground-glass lesions in both lungs and bilateral pleural effusion. We summarized a series of patient case characteristics and reviewed a case series that fulfills the criteria for hyperferritinemic syndrome and CLS reported in the literature. The patient was eventually diagnosed with RA combined with hyperferritinemic syndrome and CLS. Then she received GC (160 mg qd) combined with intravenous immunoglobulin (IVIG, 20 g/d, for 3 days). After treatment, her temperature gradually decreased, but still fluctuated. We considered that the patient also had an overwhelming proinflammatory cytokine storm, so she received a strong anti-inflammatory treatment with tocilizumab (TCZ) (400 mg qm). The patient symptoms and follow-up chest CT showed significant improvement following treatment (Fig. 8). Subsequently, the patient returned regularly, and the GC dose was gradually reduced to discontinuation according to the criteria. Following up to now, the patient was taking MTX (15 mg qw) and TCZ (400 mg qm) continuously and the disease was in sustained remission.

## 3. Discussion

Ferritin plays a role in inflammation, tumors, and neurodegenerative diseases.^[1]^ It may be pathogenic in these conditions and its reduction is expected to improve the patient condition. To date, no definitive criteria have been established for the diagnosis of the hyperferritinemic syndrome. Some scholars think that it is associated with several inflammatory conditions, such as sepsis, systemic inflammatory response syndrome, multiorgan dysfunction syndrome (MODS), macrophage activation syndrome (MAS) and catastrophic antiphospholipid syndrome.^[2]^ It is also associated with a variety of autoimmune diseases, such as SLE and RA.^[3]^ Previous evidence suggests that GC, IVIG combined with plasma exchange (PE) successfully treated the hyperferritinemic syndrome in sepsis, MODS, and MAS.^[4,5]^ And in the clinical real world, the above treatments are effective alone or in combination for these 4 diseases. Our case demonstrates the efficacy of GC combined with IVIG in the treatment of hyperferritinemic syndrome secondary to RA.

CLS is a serious complication of systemic inflammatory response syndrome characterized by the leakage of large amounts of fluid and plasma proteins into the interstitial space, resulting in hypoalbuminemia, hypovolemic shock, elevated blood concentration, systemic progressive edema, and multiple serosal cavity effusion, which can affect multiple organs throughout the body, especially the lungs and kidneys. Cases have previously reported that autoimmune diseases can be associated with CLS, such as antiphospholipid syndrome, Sjogren syndrome (SS), systemic sclerosis, and polymyositis.^[6]^ However, cases secondary to RA have not been reported. This paper fills the gap in this field and provides a reference for further research in the future. Fluid therapy is essential for the treatment of CLS, and for CLS induced by a variety of diseases, GC combined with high-dose IVIG may be an effective treatment strategy.^[7]^ In our case, the patient also had an urgent onset, severe systemic symptoms, and critical condition. Therefore, under the premise of fluid management, we gave the patient GC combined with high-dose IVIG earlier, with better efficacy.

Tocilizumab is a recombinant, fully humanized monoclonal antibody which targets both soluble and membrane-bound forms of the interleukin-6 receptor. Currently, tocilizumab is approved by the FDA for treating RA, giant cell arteritis, system sclerosis-associated interstitial lung disease, polyarticular and systemic juvenile idiopathic arthritis, and cytokine release syndrome (CRS). Our patients were treated with TCZ mainly based on the following 2 considerations: The patient diagnosis of RA was clear, and TCZ is effective in the treatment of moderate to severe active RA, which had reliable evidence-based evidence. TCZ was effective in the treatment of CRS associated with CAR-T cell therapy and CRS associated with COVID-19 infection.^[8,9]^ But this patient had an overwhelming proinflammatory cytokine storm, GC combined with high-dose IVIG therapy still could not completely inhibit the inflammatory storm in vivo. Therefore, we pioneered the use of TCZ in the treatment of hyperferritinemic syndrome and CLS secondary to RA, and finally achieved promising results.

In the last decade, the advent of novel therapeutic options including biologics and small molecules significantly improved the outcome of RA patients and, in particular, their quality of life. However, some patients who present with atypical clinical symptoms still have to be highly valued by clinicians and require careful diagnosis and treatment. For some rare complications, clinicians should actively consult the literature to refer to previous experience and identify and give appropriate treatment to patients as early as possible. In this case report, we present a case of a patient with secondary hyperferritinemic syndrome and CLS, with the aim of improving diagnostic ideas for clinicians and consequently improving the diagnosis and treatment of the hyperferritinemic syndrome and CLS.

This study also has its limitations. In this case, the patient received tocilizumab with significant effect. However, because cases of RA combined with methaproteinaemia syndrome and capillary leak syndrome are rare in clinical practice, only 1 patient was reported in this study. More studies are needed to investigate the efficacy and safety of tocilizumab in such patients. In the future, we will continue to collect data from such patients for analysis to provide more reliable evidence.

## Acknowledgments

We sincerely thank the editor and all the reviewers for their efforts.

## Author contributions

**Conceptualization:** Zhendong He, Fang Qin.

**Data curation:** Zhendong He, Hanyou Mo, Leting Zheng, Wen Zeng, Jing Wen, Zhanrui Chen, Fang Qin.

**Formal analysis:** Zhendong He.

**Investigation:** Hanyou Mo, Leting Zheng.

**Writing – original draft:** Zhendong He.

**Writing – review & editing:** Fang Qin.
